# Guipi decoction for insomnia: Systematic review and meta-analysis

**DOI:** 10.1097/MD.0000000000021031

**Published:** 2020-07-02

**Authors:** Mao Li, Rui Lan, Yong Wen, Kejin Shi, Dongdong Yang

**Affiliations:** a Hospital of Chengdu University of Traditional Chinese Medicine, Chengdu; b The First Affiliated Hospital of Henan University of Traditional Chinese Medicine, Zhengzhou, PR China.

**Keywords:** Guipi decoction, insomnia, meta-analysis, protocol, systematic review, traditional Chinese medicine

## Abstract

**Background:**

Insomnia is a common sleep disorder with symptoms including difficulty falling asleep and early awakening. Guipi decoction is widely used in clinical treatment of insomnia in China. However, there is a lack of systematic evaluation and analysis of Guipi decoction. Therefore, our study will provide efficacy assessments and adverse events assessments.

**Methods:**

A comprehensive search for randomized controlled trials of Gupi decoction treatments for insomnia will be carried in MEDLINE, EMBASE, the Cochrane Central Register of Controlled Trial (CENTRAL), CINAHL, AMED and Chinese databases include CBM, CNKI, CQVIP, and Wanfang from their inceptions to May 2020. Relevant reference lists, Baidu Scholar and grey literature will also be checked. Two experienced reviewers will independently search all databases. Primary outcomes include Pittsburgh sleep quality index and clinical effective rate, and secondary outcomes include traditional Chinese medicine syndrome, adverse events, and Epworth Sleepiness Scale. Review Manager 5.3 software will be used analyze all data.

**Results:**

This article will be dedicated to assessing the efficacy and safety of Guipi decoction for insomnia.

**Conclusion:**

The conclusion of this systematic review will provide evidence to judge whether Guipi Decoction is an effective therapeutic intervention for patient with insomnia. Maybe these results could potentially be helpful for improving the therapeutic strategy of patients with insomnia.

**PROSPERO registration number:**

CRD 42020164911.

## 1 Introduction

Insomnia is defined as a purely subjective complaint of dissatisfaction with sleep quality and quantity in spite of adequate opportunity for sleep, which is the most common sleep disorder among adults, especially affecting individuals of advanced age or with neurodegenerative disease.^[1]^ It is characterized by persistent difficulty initiating or maintaining sleep, along with associated daytime impairment.^[2]^ As much as one-third of the population experiences transient insomnia symptoms at any given time.^[3]^ For many people, insomnia is a persistent condition, with 74% reporting symptoms at least over one year.^[4]^ In China, 45.4% of people have experienced insomnia in different degrees in the past month.^[5]^ Long term insomnia not only brings great trouble to people's life and work, but also causes diseases such as depression,^[6]^ anxiety disorders,^[7]^ heart disease,^[8]^ stroke,^[9]^ hypertension,^[10]^ diabetes,^[11]^ dementia.^[12]^ Meanwhile, insomnia is associated with an increased risk of car crashes, as well as injuries at home and at work.^[13]^ Insomnia, and its comorbid problems, leads to high societal costs, mainly due to productivity losses.^[14,15]^

Generally speaking, the treatments for insomnia are divided into nonpharmacologic and pharmacologic therapies. Cognitive behavioral treatment for insomnia (CBT-I) is an evidence-based non-pharmacologic treatment with large effects on insomnia severity,^[16]^ and is recommended by international guidelines^[17]^ as the first treatment option for insomnia, offering advantages over existing pharmacotherapies with regard to safety and durability of response.^[18]^ Although the efficacy of CBT-I is still undisputed, problems with accessibility and cost effectiveness mean that many people with insomnia cannot benefit from this treatment.^[19]^ Pharmacologic interventions are often prescribed for insomnia, including benzodiazepines and other nonbenzodiazepine sedative-hypnotics. Sedative-hypnotics are categorized as high-risk medications, particularly among older adults, and are associated with preventable harm including falls, hip fractures, delirium, and death.^[20,21]^ Observational studies suggest that most indications for sedative-hypnotic initiation in hospitals are potentially inappropriate.^[22,23]^ Therefore, an increasing number of insomnia sufferers are seeking for complementary and alternative therapy for certain advantages like convenient, cheap, and less side effects.^[24]^

Chinese herb medicine has been used to manage insomnia for thousands of years. Guipi decoction is one of Chinese herb formulas, and it is widely used in clinical treatment of insomnia in China. However, there is a lack of systematic evaluation and analysis of Guipi decoction. Therefore, our study will provide efficacy assessments and adverse events assessments.

## 2 Methods

### 2.1 Protocol and registration

The protocol of this systematic review and meta-analysis has been registered on PROSPERO platform (https://www.crd.york.ac.uk/PROSPERO) with an assigned registration number CRD42020164911, basing on the Preferred Reporting Items for Systematic Reviews and Meta-Analyses Protocols statement guidelines.

### 2.2 Eligibility criteria

#### 2.2.1 Types of study

All randomized controlled trials that investigate the efficacy and safety of Guipi decoction treatments for insomnia will be included. It will be excluded if studies using inappropriate random sequence generation methods such as alternate allocation or birth day. It will be considered a randomized controlled trial and will be included in this review if a study only mentions randomization and does not explain the randomization method.

#### 2.2.2 Types of participants

Male or female patients diagnosed with insomnia are included. The diagnosis of insomnia needs to be consistent with ICSD-3 in the International Classification of Sleep Disorders II or Guidelines for the diagnosis and treatment of insomnia in China. There are no limitations on the age.

#### 2.2.3 Types of interventions

The experimental group should be treated with Guipi decoction alone or in combination with western medicine. The control group received single therapy such as benzodiazepines, nonbenzodiazepine hypnotics, placebo, or other basic treatment. Combining therapy which cannot judge the effect of acupuncture will be excluded. The treatment duration is unlimited. Nursing measures should be consistent between the 2 groups.

#### 2.2.4 Types of outcome measures

Primary outcomes: The primary outcome includes Pittsburgh sleep quality index and clinical effective rate. Pittsburgh sleep quality index is comprised of 19 self-rated items in 7 factors and the total scores range from 0 to 21.^[25]^ Clinical effective rate is calculated based on the criteria for the therapeutic effects of western medicine and traditional Chinese medicine, according to Guiding Principles for Clinical Research of New Chinese Medicine. Clinical recovery: sleep time has returned to normal or the sleep time at night has been more than 6 hours, deep sleep and energetic after waking up. Markedly effective: sleep has improved significantly, sleep time has increased by more than 3 hours, and sleep depth has increased. Effective: the symptoms have relieved, but the sleep time increases less than 3 hours longer than before. Invalid: no improvement or aggravation of insomnia. The total effective rate = (Clinical recovery + Markedly effective + Effective)/Total number of cases ×100%.^[26]^

Secondary outcomes: Syndrome according to standards for assessing traditional Chinese medicine. Adverse events caused by Guipi decoction, such as dizziness, nausea, vomiting, weariness, etc. Daytime function measured by standardized sleep-related scales, for example, the Epworth Sleepiness Scale.^[27]^

### 2.3 Data sources and search strategy

We will search MEDLINE, EMBASE, the Cochrane Central Register of Controlled Trial, CINAHL, AMED, and Chinese databases include CBM, CNKI, CQVIP, and Wanfang from their inceptions to May 2020. The language of publication will be limited to Chinese and English. But it will not limit the race of the participants. We will also check the reference lists of the relevant articles and perform a manual check on Baidu Scholar and grey literature. The searching strategies for PubMed are listed in Appendix Table 1 for details, and will be modified and used similarly for the other databases.

**Table 1:**
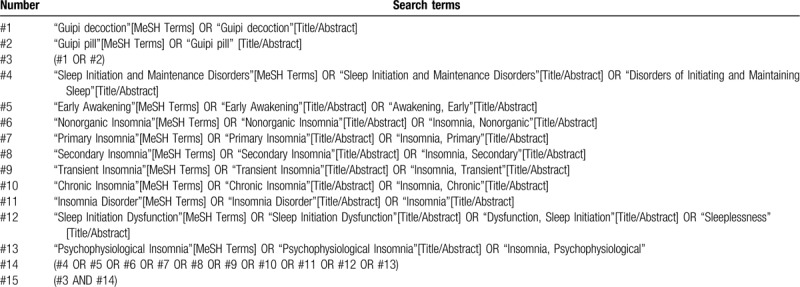
Search strategies for the PubMed.

### 2.4 Study selection and data extraction

**Figure 1:**
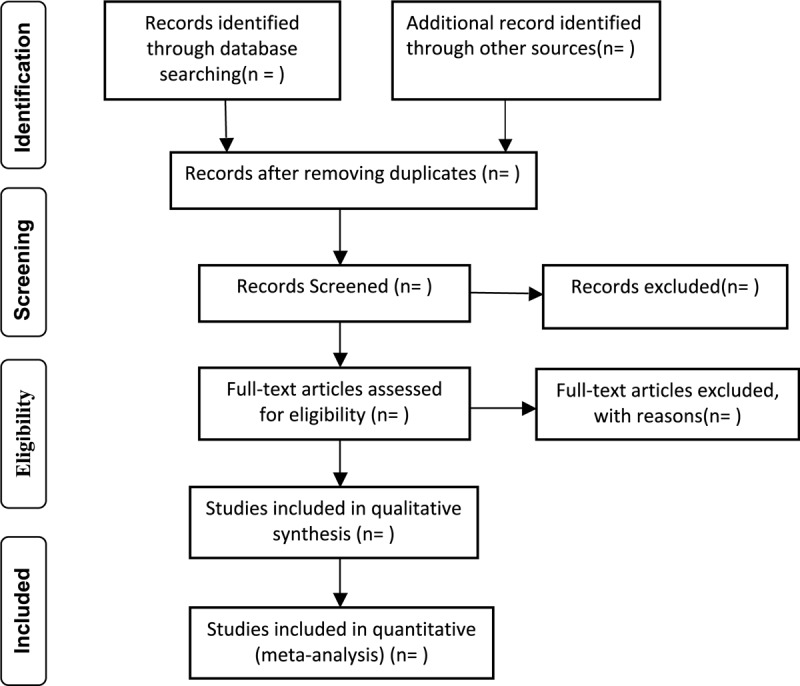
Flow chart of study selection process.

Two experienced reviewers (WY, SKJ) will independently search all databases according to the search strategies, read the titles and abstracts to select potential references, exclude obviously unrelated literature, and delete duplicated relevant research abstracts. Then, they will read the full text to assess eligible studies. If the information is incomplete, the reviewer should contact the author to obtain complete information. If there is a disagreement it will be resolved through discussion or a third reviewer (YDD). The complete selection process will be presented in a Preferred Reporting Items for Systematic Reviews and Meta-Analyses flow chart (Fig. 1).

Data extraction will be performed by 2 reviewers (WY, SKJ) independently. Information extracted from included studies will include: literature characteristics (the first author's name, journal, year of publication, country, aim, funding sources); participant information (age, gender, diagnose criteria, exclusion criteria, disease duration or stage, sample size, assessment of compliance, and withdrawals); intervention information (details of intervention and control, treatment duration); and outcome (results, adverse events, duration of follow-up, subgroup analysis).

### 2.5 Data analysis

#### 2.5.1 Risk of bias assessment

Two review authors (WY, SKJ) will independently assess the risk of bias in included studies using the Cochrane risk of bias assessment tool by considering the following characteristics:

1. Randomization sequence generation,
2. Allocation concealment,
3. Blinding of outcome assessment,
4. Incomplete outcome data,
5. Selective outcome reporting,
6. Other sources of bias.

Each domain will be divided into 3 categories: low risk of bias, high risk of bias, and unclear risk of bias. Any discrepancy in the assessment of risk of bias will be resolved by discussion and a third review author will be consulted if necessary.

#### 2.5.2 Date synthesis

Data will be analyzed using Rev Man software (Version 5.3) provided by the Cochrane Collaboration (www.cochrane.org). A meta-analysis using random or fixed effects models will be conducted to pool the data. Dichotomous data will be adopted and expressed as risk ratio. Continuous data will be pooled and presented as mean difference or standardized mean difference. The confidence interval is established at 95%.

#### 2.5.3 Assessment of heterogeneity

Heterogeneity will be examined using the *I*^2^ test (α = 0.1) according to the Cochrane Handbook (0%–40%, might not be important, 30%–60%, may represent moderate heterogeneity, 50%–90%, may represent substantial heterogeneity, and 75%–100% may represent considerable heterogeneity). If the *I*^2^ value is higher than 50%, the random effects model will be used. Otherwise the fixed effect model is involved.

#### 2.5.4 Subgroup analysis and sensitivity analysis

If heterogeneity is evaluated as significant, we will perform a subgroup analysis to explore the possible causes of heterogeneity according to the difference in participant characteristics, interventions, controls, and outcome measures. We will use sensitivity analysis to enhance the credibility of the results by eliminating studies with high risk of bias, missing data studies, and outliers.

#### 2.5.5 Assessment of reporting bias

Funnel plots will be used to evaluate the reporting biases when more than 10 trials are included. A symmetrical distribution of funnel plot data indicates that there is no reporting bias. On the contrary, an asymmetry distribution of funnel plot data implies reporting bias, and we will attempt to explain possible reasons.

#### 2.5.6 Grading the quality of evidence

We will evaluate the quality of evidence through guidelines of the Grading of Recommendations, Assessment, Development, and Evaluation (GRADE).^[28]^ The evidence quality will be ranked by 4 levels: high quality, moderate quality, low quality, and very low quality.

#### 2.5.7 Ethics and dissemination

Ethical approval will be unnecessary because the data included in this systematic review come from published literature and there will be no concerns regarding privacy. Findings of this research will be disseminated in a peer-reviewed journal or conference presentations.

## 3 Discussion

Insomnia is a common sleep disorder with symptoms including difficulty falling asleep and early awakening. With the increasingly fast pace of our life, the incidence of insomnia is also increasing. Because of time consumption and lack of adequately trained providers, and economic issues, CBT is rarely used in routine clinical practice, and pharmacotherapies for insomnia can cause serious adverse effects. Given the limitations of both CBT and pharmacological therapy, it is necessary to search for complementary and alternative therapy with higher effective rate and less side effects for insomnia. Guipi decoction originated in Song Dynasty, and has been used for more than 700 years in the treatment of insomnia. However, there is no systematic review to evaluate the efficacy and safety of Guipi Decoction for insomnia. Therefore, it is necessary to perform a high-quality systematic review and meta-analysis of it. Maybe these results could potentially be helpful for improving the therapeutic strategy of patients with insomnia.

## Author contributions

Mao Li, Rui Lan, and Dongdong Yang contributed to the conception of the study. The manuscript protocol was drafted by Mao Li and was revised by Dongdong Yang and Rui Lan. The search strategy was developed by all the authors and performed by Yong Wen and Kejin Shi, who also independently screened the potential studies, extracted data from the included studies, assessed the risk of bias, and completed the data synthesis. Dongdong Yang arbitrated in cases of disagreement and ensured the absence of errors. All authors approved the publication of the protocol.

**Conceptualization:** Mao Li, Rui Lan, Dongdong Yang.

**Data curation:** Mao Li, Kejin Shi.

**Formal analysis:** Yong Wen, Kejin Shi.

**Funding acquisition:** Rui Lan, Dongdong Yang.

**Methodology:** Yong Wen, Kejin Shi.

**Software:** Mao Li, Yong Wen, Kejin Shi.

**Supervision:** Rui Lan, Dongdong Yang.

**Writing – original draft:** Mao Li.

**Writing – review & editing:** Mao Li.
